# How Can We Improve Vaccination Response in Old People? Part I: Targeting Immunosenescence of Innate Immunity Cells

**DOI:** 10.3390/ijms23179880

**Published:** 2022-08-31

**Authors:** Anna Aiello, Mattia Emanuela Ligotti, Maider Garnica, Giulia Accardi, Anna Calabrò, Fanny Pojero, Hugo Arasanz, Ana Bocanegra, Ester Blanco, Luisa Chocarro, Miriam Echaide, Leticia Fernandez-Rubio, Pablo Ramos, Sergio Piñeiro-Hermida, Grazyna Kochan, Nahid Zareian, Farzin Farzaneh, David Escors, Calogero Caruso, Giuseppina Candore

**Affiliations:** 1 Laboratory of Immunopathology and Immunosenescence, Department of Biomedicine, Neurosciences and Advanced Technologies, University of Palermo, 90133 Palermo, Italy; 2 Oncoimmunology Group, Navarrabiomed, Instituto de Investigación Sanitaria de Navarra (IdiSNA), 31008 Pamplona, Spain; 3 Medical Oncology Department, Hospital Universitario de Navarra, Instituto de Investigación Sanitaria de Navarra (IdiSNA), 31008 Pamplona, Spain; 4 Division of Gene Therapy and Regulation of Gene Expression, Centro de Investigación Médica Aplicada (CIMA), Instituto de Investigación Sanitaria de Navarra (IdiSNA), 31008 Pamplona, Spain; 5 The Rayne Institute, School of Cancer and Pharmaceutical Sciences, King’s College London, London WC2R 2LS, UK

**Keywords:** adjuvants, aging, dendritic cells, immunosenescence, immunostimulation, innate immunity, trained immunity, vaccines

## Abstract

Vaccination, being able to prevent millions of cases of infectious diseases around the world every year, is the most effective medical intervention ever introduced. However, immunosenescence makes vaccines less effective in providing protection to older people. Although most studies explain that this is mainly due to the immunosenescence of T and B cells, the immunosenescence of innate immunity can also be a significant contributing factor. Alterations in function, number, subset, and distribution of blood neutrophils, monocytes, and natural killer and dendritic cells are detected in aging, thus potentially reducing the efficacy of vaccines in older individuals. In this paper, we focus on the immunosenescence of the innate blood immune cells. We discuss possible strategies to counteract the immunosenescence of innate immunity in order to improve the response to vaccination. In particular, we focus on advances in understanding the role and the development of new adjuvants, such as TLR agonists, considered a promising strategy to increase vaccination efficiency in older individuals.

## 1. Introduction

### 1.1. Trends in Aging: Consequences and Solutions

Thanks to the improvements in public health and medicine, as well as the economic, social, and educational developments experienced during the last century, the Western population is living longer. The worldwide population over the age of 60 will increase from 1 billion in 2020 to 1.4 billion in 2030. By 2050, the global population over 60 years of age will double [1]. However, this increase in lifespan will not be accompanied by an improvement in the quality of life [2].

Older people, in particular over 75 years, are more vulnerable to diseases, and as the population ages the pressure on health systems increases. Infections are one of the main causes of disease and/or death in people aged 65 years and over. Specifically, infectious diseases, such as influenza and pneumonia, are among the top killers. For this reason, an effective approach to improve the health of this age category is preventive medicine and vaccination [3,4]. Unfortunately, older people frequently show impaired responses to vaccines. Thus, even after conventional vaccination, an increased risk of contracting infections remains [5]. Indeed, most vaccines have been first tested in young or adult people recruited in clinical trials, with an underrepresentation of the older population. Notably, older people do not develop an adequate immune response against new pathogens or vaccines due to immunosenescence.

Immunosenescence is a highly dynamic and multifactorial process, consisting of several changes in immune responses, in which most functions decrease dramatically while others are maintained or even increased. Age-related changes affect both innate and adaptive immunity and are implicated in the increasing frequency and severity of new infections as well as a decreased response to vaccination [6,7]. Specifically, the hallmarks of the immunosenescence of adaptive immunity are thought to be a decrease in the naïve lymphocyte compartment, coupled with the accumulation of highly differentiated memory cells. Both naïve and memory cells can present functional alterations. In addition, studies on natural immunity in older people have evidenced that aging has a profound impact on the number and functions of the innate immune cells. However, no specific definition of the hallmarks of innate immunosenescence has yet been defined [6,8,9].

Immunosenescence is accompanied by a chronic, sterile, and low-grade inflammation termed inflammaging, which in turn contributes to immunosenescence [8,9]. Inflammaging is related to genetics, microbiome, visceral obesity, socio-economic status, education, pollution, and chronic infections. The latter chronically stimulates some T lymphocyte subsets to produce pro-inflammatory cytokines [10,11,12]. One of the main causes of inflammaging is the increased presence of senescent cells. These cells acquire the senescence-associated secretory phenotype, producing inflammatory mediators, thus contributing to systemic chronic low-grade inflammation [13].

Several approaches have been assessed for their efficacy in restoring immunity in older people, including vaccines with higher doses of antigens, different routes of administration, or the use of new adjuvants. Nevertheless, although stronger responses have been achieved by some of these means, the net result is still unsatisfactory [8,14]. Although nearly all studies to enhance the immune response to pathogens and vaccines target adaptive immunity, innate immunity should be also considered given its role in immunosenescence [8].

### 1.2. The Innate Immune System 

The immune system is constituted by cells and molecules which grant immunity and are able to counteract the onset of diseases caused by infectious agents or by immunogens. The immune system can be divided into two arms, innate immunity and adaptive immunity.

Innate immunity is set by cellular and biochemical responses that stop the etiological disease agents, generally rapidly and in non-specific manners. Cellular components are represented by monocytes (macrophages in the tissues), neutrophils, natural killer (NK) cells, and dendritic cells (DCs); soluble mediators are complement proteins, as well as cytokines and chemokines, which favour the regulation of the innate response and the recruitment of immune cells.

Adaptive immunity is a specific type of immunity that promotes immunological memory by recognising pathogens, for which a response had previously been developed. Adaptive immunity is represented by B and T lymphocytes and takes several days or weeks to reach the maximum response. T cells are responsible for the cellular immune response, while B cells are responsible for the humoral (antibody-based) immune responses [9,15].

Recent studies have discovered that myeloid and NK cells also have a kind of immunological memory. Exposure to some microbial components, in particular from the *Bacillus* Calmette-Guérin (BCG), trains myelomonocytic cells to develop an enhanced effector function against microbial agents [16,17,18]. This phenomenon is called trained immunity. The cellular basis of trained immunity is explained by epigenetic and metabolic reprogramming. Trained myeloid cells exhibit increased killing capacity and the production of cytokines, chemokines, and fluid-phase pattern-recognition molecules for an extended period of time following their initial activation. These cells become better suited for triggering acquired immune responses for the non-specific enhancement of biological functions [16]. However, it has yet to be explored whether trained immune responses show an age-related decrease in order to find strategies to overcome this dysregulation.

Innate and adaptive immunity interact through a dense network of interconnections. Impairment of any one of these interactions results in a reduced ability of the immune system to respond to challenges affecting health [9].

In this paper, we focus on immunosenescence of the innate immune cells and on the possible strategies to enhance the response to vaccines. Only the innate immune cells circulating in the blood will be discussed, since the vast majority of studies focus on these cells due to the greater ease of access.

## 2. Immunosenescence of the Innate Immune Cells

### 2.1. Neutrophils

Neutrophils act as phagocytic cells in the first phases of immune interaction with pathogens, with the key function of responding rapidly to chemical stimuli (chemotaxis). In the aging process, they contribute to inflammaging because of their capacity to recognize pathogen-associated molecular patterns (PAMPs), damage-associated molecular patterns (DAMPs) and/or cellular debris released after the entry of the pathogen via their pattern-recognition receptors (PRRs). In older people, this leads to an increased production of pro-inflammatory mediators and to the exacerbation of the inflammaging process [9].

In healthy young people, neutrophils have a short lifespan, which is increased by antigen challenge. Unlike other cell populations involved in immunosenescence, neutrophils do not show an obvious aging phenotype, which makes it more complicated to fully define their characteristics in the aging process [19,20]. However, aging appears to promote a dysfunctional chemotactic capacity in neutrophils, which alters both dissemination and achievement of the site of damage. This is demonstrated by phenotypic changes, such as: (i) decreased expression of CD11b, which is involved in the recruitment of neutrophils to the infected tissue; (ii) up-regulation of chemokine receptors and adhesion molecules CXCR4, CD49d, and ICAM, which facilitate neutrophil migration through the endothelium; (iii) down-regulation of chemokine receptor CXCR2 which determines the capacity to reverse-transmigrate in the vessels; (iv) decreased mobilisation of actin and calcium related to the migration capacity [21,22,23]. Defects in chemotaxis and, consequently, in the ability to reach the site of damage, lead to altered neutrophil distribution, which also impairs pathogen recognition and elimination. In addition, the non-specific distribution of neutrophils may cause tissue damage due to the release of tissue-degrading neutrophil elastases. This can be evidenced by the increased expression of both CD63 on the cell surface and the increased release of protease from granules. CD63 contributes not only to tissue damage but also to systemic inflammation related to the ability to disseminate through different organs in a trans-endothelial manner [23,24].

Furthermore, neutrophils from older people show defects in the clearance of pathogens and in wound healing. These two dysfunctions can be due to the impaired expression of toll-like receptors (TLRs) and a decreased major histocompatibility complex (MHC) class II expression, as well as increased production of reactive oxygen species (ROS) and decreased expression of CD16 (also known as FcγRIIIA, a low-affinity receptor for the Fc portion of immunoglobulin G). The latter event affects complement- and immunoglobulin-mediated opsonization of pathogens and impairs the ability of neutrophils to create the neutrophil extracellular trap (NET), a trap for microbes in the process called NET-osis [20,25]. The combination of these events contributes to an enhanced status of inflammaging and may be a possible explanation for the greater susceptibility of the older people to invasive bacterial infections [23]. The majority of these events can be detected following in vitro stimulation with formyl-methionine-leucine-phenylalanine, lipopolysaccharide (LPS), and granulocyte–macrophage colony-stimulating factor (GM-CSF) [26].

Several studies demonstrate that functional alterations of neutrophils with age are stimulus-dependent and are related to the capacity of these cells to respond appropriately to infections [27,28]. For example, it has been observed that a reduced number of neutrophils in the older people are affected by severe infections, due to a reduced capacity of neutrophil progenitors to respond to G-CSF and, consequently, to differentiate into mature neutrophils. Moreover, signalling pathway defects or a steady-state activation can lead to alterations in the migration or phagocytic capacity of neutrophils from older donors [29,30].Altered neutrophil membrane fluidity in older people and the subsequent reduction in the level of lipid rafts are associated with defects in receptor recruitment and in the downstream signalling events. This, in turn, reduces the ability of neutrophils to survive longer at the site of inflammation, limiting their ability to respond to prolonged infections. Studies have highlighted potential mechanisms for altered cell behaviours which appear to be shared across disease states. It has been suggested that altered functions may represent neutrophil “senescence” [20].

A recent study conducted in animal models has shown that the aging process impairs vaccine-mediated protection against *S. pneumoniae* due to the impaired ability of neutrophils to kill the bacteria, even when they were opsonized with immune sera from young controls [31]. In the increased incidence/development of cancer in older people, neutrophils appear to play an immunosuppressive role, interfering with the normal process of immune surveillance against cancer-associated antigens [32]. Indeed, it is possible to distinguish two neutrophil subtypes in the cancer microenvironment: (i) tumour-associated neutrophils 1 (TAN1) and (ii) tumour-associated neutrophils 2 (TAN2). TAN2 resembles aged neutrophils in their reduced chemotactic and phagocytic capacity, and their impaired free radical production and apoptosis [33].

Neutrophils have an important role in the clearance of virus infections and, in particular, in human cytomegalovirus (HCMV) infection by killing the latently infected cells via antibody-dependent cellular cytotoxicity (ADCC). However, HCMV can implement an immune evasion strategy, preventing the recruitment of neutrophils to the site of infections by inhibiting their chemoattractant-mediated recruitment, thus blocking the killing of infected cells [34].

There is also an emerging role for neutrophils in the new SARS-CoV-2 infection and this may be important for better understanding the higher incidence of COVID-19 disease in older people. Recent studies in aged rhesus macaques have shown the presence of increased G-CSF levels, associated with earlier neutrophil release from the bone marrow. These early released neutrophils are less mature, have reduced function, and some possess a unique myeloid-derived suppressor-cell phenotype [35]. In physiological conditions, high G-CSF levels in these aged animals are associated with higher levels of other proinflammatory cytokines in the context of inflammaging. These findings may partially explain the severity of COVID-19 in aged individuals, providing support for the possible targeting of G-CSF and GM-CSF in the clinical management of this pathology in older patients [35,36].

### 2.2. Monocytes

Monocytes are phagocytic cells derived from the bone marrow (BM) from hematopoietic stem cells (HSCs), from which all other innate and adaptive immune cells are derived. They are involved in innate immunity and in the response toward infections, thanks to the expression of both TLRs and PRRs and subsequent production of pro-inflammatory cytokines. In particular, in humans, the TLR family consists of 10 members expressed on the cell surface (TLR1, 2, 4, 5, 6, 10), or in the endosomal compartments (TLR3, 7, 8, 9), binding lipid-, protein-, and nucleic-acid-based TLR agonists, responsible for the recognition of bacterial and viral nucleic acids, including double- and single-stranded RNA generated through the autophagy process [37,38].

Monocytes participate in the resolution of inflammation, producing anti-inflammatory cytokines and lipidic mediators, enabling them to participate in wound healing and tissue remodelling. They also have an important role as antigen presenting cells (APCs) [28]. There are three different subsets of monocytes which can be distinguished on the basis of the expression of surface molecules: (i) classical CD14^+^CD16^−^, (ii) intermediate CD14^+^CD16^+^, (iii) and non-classical CD14^dim/low^CD16^+^. The main pattern of monocyte differentiation suggests that there is a transition from classical through intermediate to non-classical monocytes, which resemble an exhausted/senescent-like phenotype with shorter telomeres, contributing to the inflammaging status [25,39].

CD14^+^CD16^−^ monocytes have inflammatory properties, migrate between tissues and lymph nodes to present antigens and back into tissues where they can differentiate into macrophages. They can also produce pro-inflammatory cytokines and recruit other inflammatory cells. CD14^+^CD16^+^ monocytes have a high capacity to present antigen and produce tumour necrosis factor (TNF)-α and interleukin (IL)-1β after stimulation; whereas CD14^dim/low^CD16^+^ monocytes have patrolling properties. The presence of CD16 expression makes these monocytes capable of antibody-dependent phagocytosis [40].

The aging process also affects the function and subset distribution of monocytes. Their heterogeneous and highly adaptable nature and their ability to respond to pathogens and cellular debris allow them to communicate with the adaptive immune system. Therefore, their age-associated defects make them a key contributor to inflammaging. In terms of numbers, there is no evident reduction in the peripheral blood of older people and, otherwise, some studies have reported their increased number in blood, which may then differentiate into DCs. Moreover, increasing age appears to be associated with an expansion of both non-classical and intermediate monocytes [41,42].

The non-classical monocytes have an inflammatory profile. They may present impairment in the expression of HLA-DR, chemokines such as CX3CR1, or generally an altered expression of co-stimulatory proteins, defining a reduced capability to present antigens and identifying a defect to migrate toward sites of tissue damage [25]. In each monocyte subset, an age-associated dysregulation in antigen presentation, thus in their ability to induce inflammatory responses, has been highlighted. Such age-associated dysregulation is also present in other APCs, and is particularly evident in the decreased numbers and reduced function of DCs and CD34^+^ hematopoietic progenitors [43]. Monocytes may downregulate their expression of MHC class II and this is accompanied by the reduced expression of some TLRs, such as TLR1, while TLR2 expression is much better maintained. The reduced TLR expression is linked with alterations in the associated intracellular signalling, which in the case of TLR1, results in impaired mitogen-activated protein kinase (MAPK) signalling, and the reduced production of pro-inflammatory cytokines, such as IL-6 and TNF-α [44]. However, such age-associated reduction in expression and activity does not appear to be present in TLR5 [25,40,45,46].

A recent analysis has shown that the reduced type-I interferon (IFN) production in aged monocytes is due to an impairment of the cytosolic PRR responsible for the type-I IFN signalling and RIG-I expression, hence activity, linked to decreased abundance of the adaptor protein TNF-receptor-associated factor 3 and IFN regulatory transcription factors. This may be one of the reasons that older people are more susceptible to respiratory infections, as type-I IFN plays an important role in the clearance of these infections [40].

Analysis of monocytes from young and older people has shown that the percentage of monocytes producing both IL-10 and IL-12 increases with age, while no difference is detected in monocytes producing IL-10 alone and a decline is evident in the percentage of monocytes producing IL-12 [43]. The ex vivo stimulation of the monocytes of older people also shows the impaired expression of inflammatory chemokines CCL3 and CCL5 following LPS stimulation. However, the data about the age-associated alterations in the expression of cytokines are controversial due to the different subpopulations and number of subjects studied, as well as differences in the study methods employed [47].

The heterogeneity in the phenotypes and function of monocytes may also be affected by the general health and frailty of the subjects studied. For example, monocytes isolated from frail older adults appear to have increased inflammatory-associated responses to LPS mediated in vitro stimulation [40].

In order to gain a clearer picture of age-associated changes in the function and phenotype of immune cells, it is important to consider changes in the mechanisms that regulate gene expression, such as epigenetic modifications, including DNA methylation of cytosines in CpG dinucleotides and histone modifications. A study of cis-gene expression-associated methylation sites (age-eMS) in CD14^+^ monocytes from older subjects shows that age-eMS tend to be hypomethylated. These hypomethylated sites appear to be preferentially located in the predicted enhancer regions that are linked to the expression of antigen processing and presentation genes (MHC class I and II). Thus, this study demonstrates an age-associated upregulation of MHC class II antigen presentation signalling pathways, which appears to be due to the elevated expression of MHC class II on the surface of monocytes and therefore with the development of age-related chronic inflammatory and autoimmune disorders related to MHC class II signalling [48].

The contribution of monocytes to the enhanced inflammatory status of older people may be also correlated with their metabolism and to the decrease in mitochondrial respiratory capacity in classical monocytes. Since mitochondrial dysfunction is impaired, it is reasonable to assume that many cell types in older individuals must produce more energy through non-oxidative metabolism, and that this could be linked to an increased basal glycolysis and even to a pro-inflammatory status. However, the ex vivo stimulation of subsets of monocytes with LPS did not demonstrate an alteration in mitochondrial metabolism, but rather the altered expression of a subset of cytokines. This may be because normal conditions may not be severe enough to demonstrate age-associated impairments in mitochondrial metabolism in monocytes. The use of trifluoromethoxy carbonyl cyanide phenylhydrazone, an uncoupler of mitochondrial oxidative phosphorylation, decreasing mitochondrial respiratory capacity, may provide sufficiently severe stress in mitochondrial function to allow the detection of impaired functions in the in vitro analysis of monocytes [47,49].

Another important issue for aging and for the characterization of immunosenescence is the lifelong interactions of immune cells with pathogens. Monocytes, together with hematopoietic progenitor cells, represent the main reservoir for HCMV latency and the main cell type in the blood that could be infected. Furthermore, monocytes favour virus reactivation through differentiation [50]. Ex vivo studies demonstrate changes in the secretome of the aged CD14^+^ monocytes and their increased susceptibility to infection [51]. However, HCMV status does not appear to influence the number and the subset distribution of monocytes in older people [52]. Instead, there is an alteration in the expression of some surface markers. Increased expression of CD163, the high affinity scavenger receptor for the haemoglobin–haptoglobin complex, correlates with increased age in women, while the expression of CD64, the Fc-gamma receptor 1, increases in relation to age and HCMV positivity [52].

A recent paper, which profiled blood mononuclear cells from healthy men and women by carefully matching their age, has demonstrated that, although the CD16^+^ monocytes increase with age in both sexes, monocyte-specific loci appear to be more activated in men than in women [53].

### 2.3. Dendritic Cells

DCs are professional APCs, specialised in the uptake, processing, and presentation of antigens to T cells. In their immature state, DCs are ubiquitously distributed in peripheral tissues, in most non-lymphoid organs and in epithelia, with a role as sentinels characterised by high phagocytic capacity and low expression levels of MHC and costimulatory molecules. In this immature state, DCs are poorly immunogenic. Upon the triggering of PRRs by pathogen-derived products or inflammatory stimuli, DCs engage a maturation program into fully functional APC, characterised by several morphologic, phenotypic, and functional changes, with down-regulation of the phagocytic capacity and upregulation of costimulatory molecules, MHC, and chemokine receptors (e.g., CCR7) as well as the secretion of cytokines. Mature resident DCs migrate through afferent lymphatics to T-cell zones of lymphoid tissues where they present processed antigens within their surface-expressed MHC molecules to naïve T lymphocytes, generating an antigen-specific response [54,55]. Among PRRs, TLRs play an important role in DC activation, being capable of sensing a wide range of organisms, from bacteria to fungi, protozoa, and viruses [56].

DCs are historically classified into myeloid DCs (mDCs), specialised in antigen processing and presentation to both CD4 and CD8 T cells. mDCs secrete high levels of IL-12 and type-III IFN. Plasmacytoid DCs (pDCs) produce high levels of IFN-α/β, critical for antiviral responses [57,58]. DC subsets show different patterns of expression of TLRs with different effects on activation. For example, pDCs express high levels of TLR7 and TLR9, the sensors of ssRNA and unmethylated CpG motif-containing DNA, respectively. The signalling pathways associated with the activation of TLRs are different with distinct biological responses. In this way, DCs can modulate the innate response, based on the type of pathogen encountered, even if TLRs are expressed on all the innate immune cells, including macrophages, neutrophils, and NK cells, on both CD4 and CD8 T cells, as well as non-immune cells such as fibroblasts and epithelial cells [59,60,61].

The published data on age-related effects on human DCs are controversial but the most commonly reported alterations concern function, number, subsets, and distribution of DCs which, in turn, lead to the reduced triggering of immune responses in older people [15,62,63]. Several studies have reported an age-associated decline in cell number and function, especially in pDC, while other reports have shown a decline with age in the number of mDC, but not of pDC [43,64,65,66,67,68]. Impaired migration and phagocytosis, and a diminished or delayed migration potential toward lymphoid organs and peripheral tissues by the aged DCs have also been reported, leading to decreased antigen presentation [23,69].

In addition, a different expression and an impairment in the function of some TLRs in older people have been observed, leading to altered TLR-mediated immune responses. For example, TLR3 and TLR8 expression in mDCs and TLR7 and TLR9 expression in pDCs appear to decline with increasing age [69,70]. Instead, TLR2 and TLR4 surface expression in mDCs appear to be unchanged in healthy older people, accompanied with detection of increased, decreased, or comparable production of TNF-α, IL-6, and IL-12 by aged mDCs in response to TLR stimulation [71,72]. These differences probably reflect differences in the assay systems and experimental conditions employed. The mechanisms underlying these alterations remain incompletely understood.

Either due to a reduction in their frequency or due to reduced expression of TLRs on mDC and pDC subsets, the antigen uptake, migratory capacity and response of DC to infections appear to be compromised with age, contributing directly or indirectly to the impaired ability of older people to respond to vaccination [73,74].

### 2.4. Natural Killer Cells

NK cells are large granular lymphoid cells, developed from common lymphoid progenitors (CLPs) and originally identified based on their intrinsic ability to lyse tumour cells without prior sensitization (hence their name) [75,76]. Defined as sentinels of innate immunity, NK cells play an important role in the host’s first line of defence against viral and tumour targets, providing immune surveillance and resistance to infection through the release of diffusible factors and cell-to-cell contact-mediated interactions. Evidence of the importance of NK cells in viral clearance and cancer control prior to the induction of adaptive immune responses has been provided by the association between NK cell deficiency and the increased incidence of viral infections and cancer [77]. NK cells also contribute to the clearance of senescent cells, which tend to increase with age, and can interact with macrophages and DCs to regulate their activation and function [78,79]. An example of NK cell interaction with other immune cells is represented by the bidirectional cross-talk with DCs, leading to the positive feedback that results in the activation of both cell types [80,81].

Consistent with their role in immune surveillance, NK cells are widespread throughout lymphoid and non-lymphoid tissues, but constitute just 10–15% of circulating lymphocytes in healthy adults. Based on phenotypic and functional features, distinct NK cell subsets have been defined in humans. Specifically, NK cells are classically identified by the expression of the cell-surface marker CD56 (also known as neural cell adhesion molecule) and the absence of the lineage marker of T cells, CD3. Based on their expression levels of the cell-surface markers CD56 and CD16, they can be divided into two subsets. The CD56^lo^CD16^+^ subset constitutes 90% of blood NK cells and is mainly responsible for natural cytotoxicity by releasing cytoplasmic granules containing the pore-forming protein perforin and the serine protease granzymes B, which induce infected cell apoptosis by caspase-dependent and -independent pathways [82]. After direct contact with target cells, this subset also secretes cytokines, such as IFN-γ. By contrast, the CD56^hi^CD16^−^ subset constitutes the remaining part of the circulating NK cells. This subset, which is particularly abundant in lymph nodes, shows minimal cytotoxic activity but has a supporting role by predominantly secreting chemokines and cytokines, including IFN-γ, TNF-α, and GM-CSF. These cytokines can modulate the function of other innate and adaptive immune cells [82]. Some studies have suggested that CD56^hi^CD16^−^ NK cells are immature precursors to mature CD56^lo^CD16^+^ NK cells, while other studies have suggested that they belong to two separate lineages [83,84]. In the linear developmental model, in the presence of inflammatory stimuli, CD56^hi^CD16^−^ NK cells progressively reduce the expression of the CD56 marker. These cells acquire the expression of the senescent marker CD57 (CD56^hi^ → CD56^lo^CD57^−^ → CD56^lo^CD57^+^). Single-cell RNA-sequencing has expanded this classical model, identifying two subpopulations among CD57^+^ NK cells [85]. The accumulation of CD57^+^ NK cells with aging has been associated with HCMV seropositivity, similar to what has been observed for CD8 T cells [86]. Recently, several studies have provided compelling evidence for the presence of memory and memory-like properties in NK cells, including long-term persistence and enhanced functional responsiveness, similar to adaptive memory T cells [87,88]. First reported in murine models, it was shown that NK cells can display vaccination-dependent, antigen-specific recall and long-lived immunological memory responses, all hallmarks of adaptive immune responses. Exposure to viral antigens or active vaccination induces epigenetic reprogramming in NK cells, making them more responsive to a secondary challenge, despite lacking antigen specificity [89]. Furthermore, BCG vaccination induces a significant increase in proinflammatory cytokine production by NK cells in response to both mycobacteria and unrelated pathogens in healthy volunteers [17]. This phenomenon, called trained immunity, makes NK cells more mature and increases their expression of cytotoxic, homing, and adhesion molecules, making them appear to have an adaptive response. This opens a new way in vaccinology to develop vaccine formulations that direct NK cells towards memory differentiation during the initial priming [90].

The cytotoxic and secretory functions of NK cells are tightly regulated by the balance of activating and inhibitory signals from an arsenal of membrane receptors, including killer cell immunoglobulin-like receptors [91]. Each NK cell expresses its repertoire of both inhibitory and activating receptors and the integration of their signals dictates their response. MHC-I molecules, expressed on the surface of almost all normal healthy cells, are ligands for NK inhibitory receptors. In the presence of this ligand–receptor interaction, the cell activation is inhibited. In contrast, infected or malignantly transformed cells downregulate MHC-I molecule expression, leading to lower inhibitory signals in NK cells, enabling the NK-mediated elimination of these cells [92]. Moreover, NK cells can be activated by several cytokines, including IL-12, IL-15, IL-18, and type-I IFNs, during the initial phases of viral infection [93].

It is well established that aging is accompanied by changes in NK cell count, phenotype, and function, linked to the risk of several diseases and infections [94]. However, there is some disagreement regarding the effect of aging on NK cell numbers, probably due to the different surface markers used to quantify them [92]. Nevertheless, it is generally accepted that aging causes an increase in both the frequency and number of CD3^−^CD56^+^ NK cells [95,96,97]. However, these cells appear to have a lower proliferation potential [98]. The increase in CD57 expression suggests a shift towards a terminally differentiated phenotype, especially in relation to HCMV infection, which is highly cytotoxic and produces high levels of IFN-γ but shows lower proliferative capacity and lower responses to cytokines [89].

Regarding the subpopulations of NK cells, aging is associated with an impaired CD56^hi^/CD56^lo^ ratio, in particular with a decreasing fraction of cytokine-producing CD56^hi^ NK cell subset, probably due to limited production of its precursors, and with an expansion of cytotoxic CD56^lo^ NK cells [86,98]. At the single-cell level, CD56^lo^ NK cells show a lower lytic capacity with age, but their greater number compensates for this numerical decrease. Decreased expression of cytotoxicity-activating receptors NKp30 and NKp46 has been observed with aging, while there are discrepancies in the expression levels of NK inhibitory receptors [99]. Although this remodelling of NK cell subsets may be a consequence of a compensation mechanism to reduce cellular cytotoxicity, it cannot be excluded that the decline of CD56^hi^ NK cells may also contribute to the development of immunosenescence because cytokines produced by this population are important for the activation of DCs and to promote inflammation as a consequence of interacting with monocytes [100,101]. For example, activated NK cells secrete TNF-α, IL-8, and IFN-γ that amplify ongoing innate immune responses and influence the early phases of the adaptive immune response. The reduced secretion of these immune-modulatory cytokines can lead to a decreased NK cell-mediated enhancement of macrophage function, with a consequent reduction in antimicrobial immunity, increased susceptibility to intracellular pathogens, and reduced NK-DC crosstalk, with impaired DC maturation, T-cell polarisation and reduced efficacy of vaccination. The decreased cytotoxic activity of NK cells observed with aging leads to an impaired killing of infected cells, with increased incidence/severity of infections [71]. This reduced lytic capacity also affects the direct elimination of senescent cells. Indeed, the elimination of senescent cells mediated by NK cells declines with advancing age, leading to the accumulation of these cells in the aged tissue and organs, thus hindering tissue homeostasis [92].

An overview of the age-associated changes in innate immunity is presented in Table 1.

## 3. Strategies to Reverse Immunosenescence of the Innate Immunity System in Older People

Immunosenescent cells show altered signalling pathways that compromise their function, having consequences on the outcomes of vaccines or other approaches to immunisation. Although most studies explain that this process is mainly due to immunosenescent T and B cells, immunosenescence in innate immunity can also be considered. Indeed, there is an increasing interest in the development of therapeutic and preventive strategies targeting innate immunity in older people.

Here, we will review the potential options for reversing immunosenescence in the innate immune system.

### 3.1. Neutrophils 

Some authors have reported the possibility of reverting the dysfunction of aged neutrophils. The treatment of aged neutrophils with statins, which act on CDC42, a cell division control protein 42 homolog, involved in the signalling to actin, improves neutrophil chemotaxis and reduces NET-osis [23]. Other reports show that high doses of simvastatin can influence the GTPase activity, involved in polarity and migration capacity. Inhibition of PI3K also appears to correct the aberrant migration seen in aged neutrophils [23]. In fact, PI3K is involved in the migration, degradation, and phagocytosis of neutrophils and its increased activity favours neutrophil-mediated damage to healthy tissue. Pharmacological activation of Nrf2, nuclear factor (erythroid-derived 2)-like 2, a transcription factor that regulates the expression of the antioxidant protein, by sulforaphane (a potent activator of Nrf2), has been shown to improve the phagocytosis of bacteria [25].

Another way to restabilize neutrophil function is to act on nutrition intake. Indeed, it has been demonstrated that the insufficient intake of some micronutrients impacts the activity of neutrophils. For example, zinc deficiency influences the development of neutrophils, vitamin E intake improves chemotaxis, and eicosapentaenoic acid intake seems to decrease the burst of ROS in older people [104,105]. Instead, the intake of copper and vitamins C and A contribute to the maintenance of neutrophil functions, while iron supports their microbicidal activity [106]. Long-term physical exercise also contributes to reducing immunosenescence and thus enhancing neutrophil function [8].

### 3.2. Monocytes

Monocytes participate in innate immunity and thus act in the early stages of an infection to promote the development of adaptive immunity through antigen presentation. They are therefore potential targets for interventions to stimulate immune responses in the older subjects. Indeed, increased expression of genes related to inflammation in the monocytes of older individuals contributes to a reduced response to vaccination. This may be due to inflammaging and immunosenescence, correlated negatively with the production of vaccination-specific antibodies [8]. Additionally, an increase in the intermediate and classical monocytes was observed after administration of a trivalent influenza vaccine, which then tended to decrease to baseline or below, accompanied by increased expression of CCR5, which correlates with the presence of an inflammatory state [107]. This indicates a potential link between the basal state of the immune system in the older people, represented by a probable increase in the number of monocytes and the inflammatory state, as well as reduced reactivity to vaccination. Interestingly, it has recently been demonstrated that monocytes from older adults, recruited to a site of inflammation or tissue damage, have a reduced ability to resolve inflammation compared to monocytes from young people [108].

CCR2^+^CD14^+^ monocytes, probably belonging to the classical and intermediate phenotype, are recruited to sites of antigen injection by the expression of CCL2 by senescent fibroblasts in the skin. CCL2 receptors (CCR2 and CCR4) are expressed by monocytes. The latter produce PGE2, which results in the inhibition of the recruitment of tissue-resident memory T-cells and thus of the antigen-specific immune response. The use of inhibitors of the p38-MAPK signalling pathway or other COX-2 inhibitors appear to restore the immune response [108,109]. This opens up new strategies to improve the immune response in older subjects.

It is possible to regulate the expression of genes related to inflammation through food intake using nutraceutical or other micronutrients, including genes in the monocyte compartment. For example, the consumption of cocoa polyphenols, rich in flavonoids, reduces the expression of adhesion molecules and pro-inflammatory markers on monocytes, such as CD40, CD36, VTLA-4, ICAM-1, or P-selectin [8]. Physical activity also influences the molecular and functional mechanisms underlying major age-related diseases, by promoting successful aging and thus longevity. In fact, long-term exercise seems to determine a reduction in inflammatory monocyte subsets and of pro-inflammatory cytokines produced, thus reducing the pace of immunosenescence [23].

### 3.3. Natural Killer Cells

NK cells play an important role as a bridge between innate and adaptive immunity [110]. This is a crucial role in the early stages of viral infection. Therefore, the ability of NK cells to exhibit adaptive-like properties make them suitable targets for strategies to improve the performance of the aged immune system. As the rate of its decline can be influenced by environmental factors, both lifestyle modifications and pharmacological interventions are candidate approaches to reduce NK immunosenescence.

Regular physical activity modulates the immune response by exerting effects dependent on duration, intensity, and type of effort applied. During physical activity, NK cell frequencies increase, probably as a consequence of the decreased presence of adhesion molecules induced by catecholamines. This exercise-mediated mobilisation of NK cells seems to be very rapid and more pronounced than that seen in T and B cells, with a maximal mobilisation in about 30 min of exercise. However, prolonged physical activity exceeding three hours leads to a decrease in circulating NK cell numbers, reflecting the migration of the mobilised NK cells to sites of muscle injury. Furthermore, factors capable of inducing their differentiation and enhancing NK cytotoxic activity are released during exercise. Among them, IL-15, highly expressed in muscle tissue and positively regulated by exercise, activates NK cell cytotoxicity through the trans-presentation of the IL-15/IL-15Rα complex on DCs [111,112].

Several lines of evidence suggest that specific dietary components have an impact on NK-cell frequencies and functions. In particular, zinc supplementation partially restores NK lytic activity, while zinc deficiency correlates with a reduction. Vitamin B9 and B12 supplementation also increase NK cell counts and activity [92].

### 3.4. Dendritic Cells

A promising strategy to increase vaccine efficiency in older adults seems to be the use of TLR agonists as adjuvants. As previously mentioned, TLRs are type-I transmembrane glycoproteins localised to the cell surface or intracellular compartments of APCs, including macrophages and DCs, but also of B and T cells. The activation of all TLRs, except for TLR3, leads to the activation of the transcription factor NF-κB through the adapter protein myeloid differentiation primary response 88 (MyD88)-dependent pathway. Following this, this signalling stimulation induces the expression of pro-inflammatory cytokine genes, such as TNF-α, IL-6, and IFN-γ, as well as the up-regulation of costimulatory molecules, including CD80, CD86, and MHC molecules [113]. TLR3 activation, instead, recruits an adapter molecule TIR-domain-containing adapter-inducing interferon-β (TRIF), activating an alternative pathway that culminates in the activation of the transcription factor interferon-regulatory factor (IRF)-3, which is pivotal for the induction of type-I IFNs [61,114]. Although TLRs are traditionally associated with innate immune cells, APCs express them at high levels. In addition, the T-cell intrinsic expression of some TLRs appears to be essential for the optimum induction of immunity [115]. For example, TLR2, TLR3, TLR5, and TLR9 act as co-stimulatory receptors to enhance the proliferation and/or cytokine production by TCR-stimulated T lymphocytes. The T-cell expression of TLRs is related to the differentiation state of different T-cell subsets. Activated/memory T cells, but not naïve CD4^+^ T cells, express appreciable levels of cell-surface TLR2 and TLR4. These data indicate that the ligand-mediated stimulation of some TLRs may contribute directly to the activation and maintenance of memory T cells [116]. Similarly, B cell expression of TLRs appears to be involved in B-cell differentiation and activation, as well as the induction of antigen-specific antibody responses [117].

The efficacy of TLR agonists as vaccine adjuvants is mainly based on their effects on DCs, as they promote antigen uptake, presentation, and maturation of DCs, as well as their cytokine secretion and thus T-cell activation [114]. Different TLR agonists are currently used in licensed vaccines as adjuvants and others are being developed for this purpose. The TLR4 agonist 3-O-desacyl-40 -monophosphoryl lipid A (MPL), a detoxified derivative of LPS extracted from Salmonella minnesota, is a part of AS01 adjuvant system, containing liposomes and the saponin QS-21, extracted from Quillaja saponaria Molina [118]. The AS01 adjuvant system was first formulated for malaria vaccination and then for a recombinant varicella zoster vaccine. Due to the synergistic action of its components, this adjuvant system effectively promotes CD4^+^ T-cell-mediated immune responses by exerting rapid and transient effects [119]. MPL is also part of the AS04 adjuvant system, in combination with aluminium salts, formulated as an adjuvant for vaccination against hepatitis B (licensed in 2005), and was then used in a human papillomavirus vaccine (licensed in 2007). In both vaccines, AS04 has induced a greater uptake of antigens by APCs and higher levels of antibodies than the same vaccine with just alum as the adjuvant [118]. The presence of alum in the AS04 formulation seems to prolong the MPL-mediated cytokine response by DCs [120]. In this case too, both components (MPL and alum) synergize, ensuring a more effective response than can be induced by either alone. CpG-1018 is a synthetic form of single-stranded DNA containing CpG dinucleotides that mimic bacterial and viral genetic material. Acting as a TLR9 agonist, CpG was used as an adjuvant in an HBV vaccine (patented in 2017) inducing a higher seroprotective response compared to an alum-adjuvanted HBV vaccine [121]. Thus, due to their ability to enhance both innate and adaptive immune responses, TLR agonists are highly promising vaccine adjuvants. However, an important consideration for their use in older individuals is the impact of aging on TLR expression levels [15]. This limitation could be overcome by the use of appropriate combinations of TLR agonists. A new type of adjuvant formulation that could enhance vaccine efficacy in the older people consists of combined adjuvants for synergistic activation of cellular immunity (CASACs). Typically, the CASAC formulation incorporates two TLR agonists, for instance CpG-oligodeoxynucleotides (a TLR9 agonist) and polyI:C (a TLR3 agonist), in combination with IFN-γ and the antigen. The latter may be individual or combinations of peptides for direct presentation by MHC-class I and II molecules or longer peptides and proteins for uptake, processing, and presentation [122]. Both are often given in a formulation of oil-in-water emulsion. The combined use of such immune stimulatory factors generates much stronger antigen-specific CD8^+^ T cell responses in aged immune-senescent mice [123]. To assess whether these promising data obtained in models can be extended to humans, the ability of two combined TLR ligands, the lipid monophosphoryl A (TLR4 agonist) and imidazoquinoline R848 (TLR7/8 agonist) to improve activation of DC isolated from older and younger healthy donors, has been examined. The stimulation of DCs with the combination of TLR4 and TLR7/8 agonists caused higher production of TNF-α and IL-12p40 in both groups, confirming the role of TLR agonists in enhancing the innate response. However, there was a 5- to 10-fold increase in the production of IL-12/p40 by the mDCs, as well as a greater amount of TNF-α by the mDCs and pDCs, when the production of these cytokines by cells from healthy older donors was compared to the production by cells from healthy younger donors [124].

**Figure 1:**
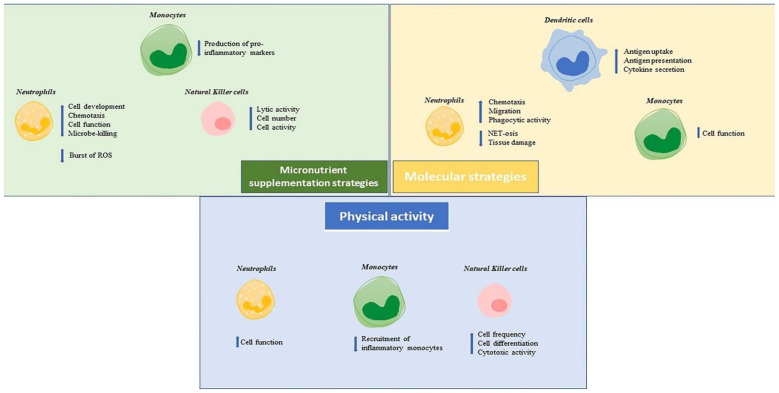
The figure shows how the main strategies to enhance the immune system response act on each cell of innate immunity.

For an overview of some approaches cited in paragraph 3, see Figure 1.

## 4. Conclusions

Although most studies explain the role of immunosenescence in the reduced efficacy of response to vaccination with a focus centred on T and B cells, innate immunity also plays an important role. In particular, alterations in the function, number, subset, and distribution of neutrophils, monocytes, natural killer cells, and DCs are detected in aging. In particular, DC changes lead to the reduced stimulation of the immune response as demonstrated in the study of a live-attenuated vaccine against yellow fever (YF) virus in the United States. The vaccination of volunteers against YF allowed the evaluation of their responses against new antigens, since the virus does not circulate in the United States. Both the acute humoral and the acute CD8^+^ T cell responses were clearly affected by aging. Low in vivo numbers of naïve T cells and peripheral DCs correlated well with reduced acute responsiveness and altered long-term persistence of human cellular immunity to YF vaccination. Thus, changes in DC function/number can play a role in the decreased response of older people to new antigenic stimulation [62].

Few interventions have directly examined the role of innate immunity in the response to vaccination in older people. The micronutrient strategies, such as zinc and vitamin E supplementation, enhance the innate immune-cell functions, improving cell development, chemotaxis, and microbicidal and lytic activities, as well as reducing different proinflammatory markers. The same goals were achieved by practising regular physical exercise, inducing a lower recruitment of inflammatory monocytes as well as increased differentiation and cytotoxic activity of NK cells. The best improvements in response to vaccination in older people could be achieved by the stimulation of pathways that are involved in key functions of the innate immune responses, such as chemotaxis and phagocytosis. The use of statins can improve the migratory capacity of neutrophils by acting on CDC2, on inhibitors of PI3K or on activators of Nrf2 that can enhance the phagocytic activity of these cells; inhibitors of p38 MAPk can improve the immune response in monocytes. Another approach may be represented by the use of molecules acting on cell receptors such as TLRs. In murine models, the simultaneous and synergistic activation of TLRs with the CASAC adjuvant has enhanced DC activation, resulting in increased cellular immune responses to vaccination. These approaches provide promising strategies to increase vaccine efficacy by aged DCs. Further studies are needed to confirm these data in humans.

## Figures and Tables

**Table 1:** Age-associated changes in innate immunity cells.

Cell	Phenotype	Changes	References
Neutrophils	CD16^+^	Reduced response to chemotactic signals; Reduced apoptosis; Impaired signal transduction; Decreased superoxide production; Decreased MHC-expression; Reduced recruitment into lipid rafts.	[20,21,22,23,25,29,30]
Monocytes	Classical (CD14^++^/CD16^−^) Intermediate (CD14^++^/CD16^+^) Non-classical (CD14^+^/CD16^++^)	Reduced absolute number and frequency of classical monocytes, increased presence of non-classical and intermediate monocytes; Reduced phagocytosis and chemotaxis; Decreased MHC expression and signalling; Decreased ROS and cytokine production; Altered TLR expression (decreased except for TLR5) and compromised function.	[25,44,45,46,47,48,49]
Dendritic cells	Myeloid (CD11c^+^/CD123^−^) Plasmacytoid (CD11c^−^/CD123^+^)	Decline in function and (or not) number of pDCs Decline in the number of mDCs Impaired migration Decreased maturation and antigen presentation; Reduced phagocytosis in mDCs; Altered TLR expression and signalling in pDCs; Altered CD80 and CD86 expression.	[23,43,57,64,65,66,67,68,69,70,71,72]
Natural Killer cells	Cytotoxic (CD56^lo^/CD16^+^) Secreting-cytokines (CD56^hi^/CD16^−^)	Decreased fraction of CD56^hi^ NK subset, expansion of cytotoxic NK subset; Decreased cytokine production by CD56^hi^ NK; Decreased lytic capacity of CD56^lo^ NK.	[86,97,98,100,102,103]

The table shows the age-associated changes in belonging to the innate immunity. The cells are classified considering the phenotype identified by the absence or presence of cluster of differentiation molecules. Abbreviations: CD, cluster of differentiation; MHC, major histocompatibility complex; ROS, reactive oxygen species; TLR, toll-like receptors; DCs, dendritic cells; NK, natural killer.

## Data Availability

Not applicable.
